# Language in Behavioral Variant Frontotemporal Dementia: Another Stone to Be Turned in Latin America

**DOI:** 10.3389/fneur.2021.702770

**Published:** 2021-08-10

**Authors:** Amandine Geraudie, Mariano Díaz Rivera, Maxime Montembeault, Adolfo M. García

**Affiliations:** ^1^Neurology Department, Toulouse University Hospital, Toulouse, France; ^2^Memory and Aging Center, Department of Neurology, University of California, San Francisco, San Francisco, CA, United States; ^3^Cognitive Neuroscience Center, Universidad de San Andrés, Buenos Aires, Argentina; ^4^Agencia Nacional de Promoción Científica y Tecnológica, Buenos Aires, Argentina; ^5^National Scientific and Technical Research Council (CONICET), Buenos Aires, Argentina; ^6^Faculty of Education, National University of Cuyo, Mendoza, Argentina; ^7^Global Brain Health Institute, University of California, San Francisco, San Francisco, CA, United States; ^8^Departamento de Lingüística y Literatura, Facultad de Humanidades, Universidad de Santiago de Chile, Santiago, Chile

**Keywords:** behavioral variant frontotemporal dementia, language, Latin America, cognitive markers, dimensional approach

## Abstract

Beyond canonical deficits in social cognition and interpersonal conduct, behavioral variant frontotemporal dementia (bvFTD) involves language difficulties in a substantial proportion of cases. However, since most evidence comes from high-income countries, the scope and relevance of language deficits in Latin American bvFTD samples remain poorly understood. As a first step toward reversing this scenario, we review studies reporting language measures in Latin American bvFTD cohorts relative to other groups. We identified 24 papers meeting systematic criteria, mainly targeting phonemic and semantic fluency, naming, semantic processing, and comprehension skills. The evidence shows widespread impairments in these domains, often related to overall cognitive disturbances. Some of these deficits may be as severe as in other diseases where they are more widely acknowledged, such as Alzheimer's disease. Considering the prevalence and informativeness of language deficits in bvFTD patients from other world regions, the need arises for more systematic research in Latin America, ideally spanning multiple domains, in diverse languages and dialects, with validated batteries. We outline key challenges and pathways of progress in this direction, laying the ground for a new regional research agenda on the disorder.

## Introduction

Behavioral variant frontotemporal dementia (bvFTD) is the most frequent form of frontotemporal dementia, a disease that affects between 1.2 and 1.8% of Latin American residents above age 55 (1). Patients exhibit insidious changes in personality and behavior, typically manifested as disinhibition, compulsion, apathy, hyperorality, and loss of empathy, alongside executive deficits and spared memory and visuospatial skills (2, 3). These domains have been the focus of neurocognitive studies on the disease, producing rich theoretical and clinical insights (4, 5). However, research on these predominant alterations has progressed to the detriment of less salient but still pervasive and debilitating impairments. Such is the case of language deficits.

Except for stereotypy of speech, difficulties with language production and comprehension are unmentioned in current international consensus criteria for bvFTD (3). These are also downplayed in overviews of the disease, which briefly present language as a widely preserved domain (6–8). Yet, several linguistic skills may be disrupted in bvFTD (9). For example, in a large group (10), naming deficits are as frequent as hyperorality (a core diagnostic feature) in the sample informing Rascovsky et al.'s criteria (55%). Moreover, specific language deficits often co-occur with typical bvFTD symptoms (11) and they can be observed even in pre-clinical stages (12). Also, despite lower severity, they may also resemble linguistic deficits in primary progressive aphasia (PPA) in their manifestation (13, 14) and progression rate (15). In addition, canonical atrophy patterns in bvFTD (2, 16) overlap with language-preferential regions, including the frontal, insular, cingulate, and temporal cortices (17–20). Thus, the neglect of language characterization in bvFTD research seems unwarranted.

**Figure 1:**
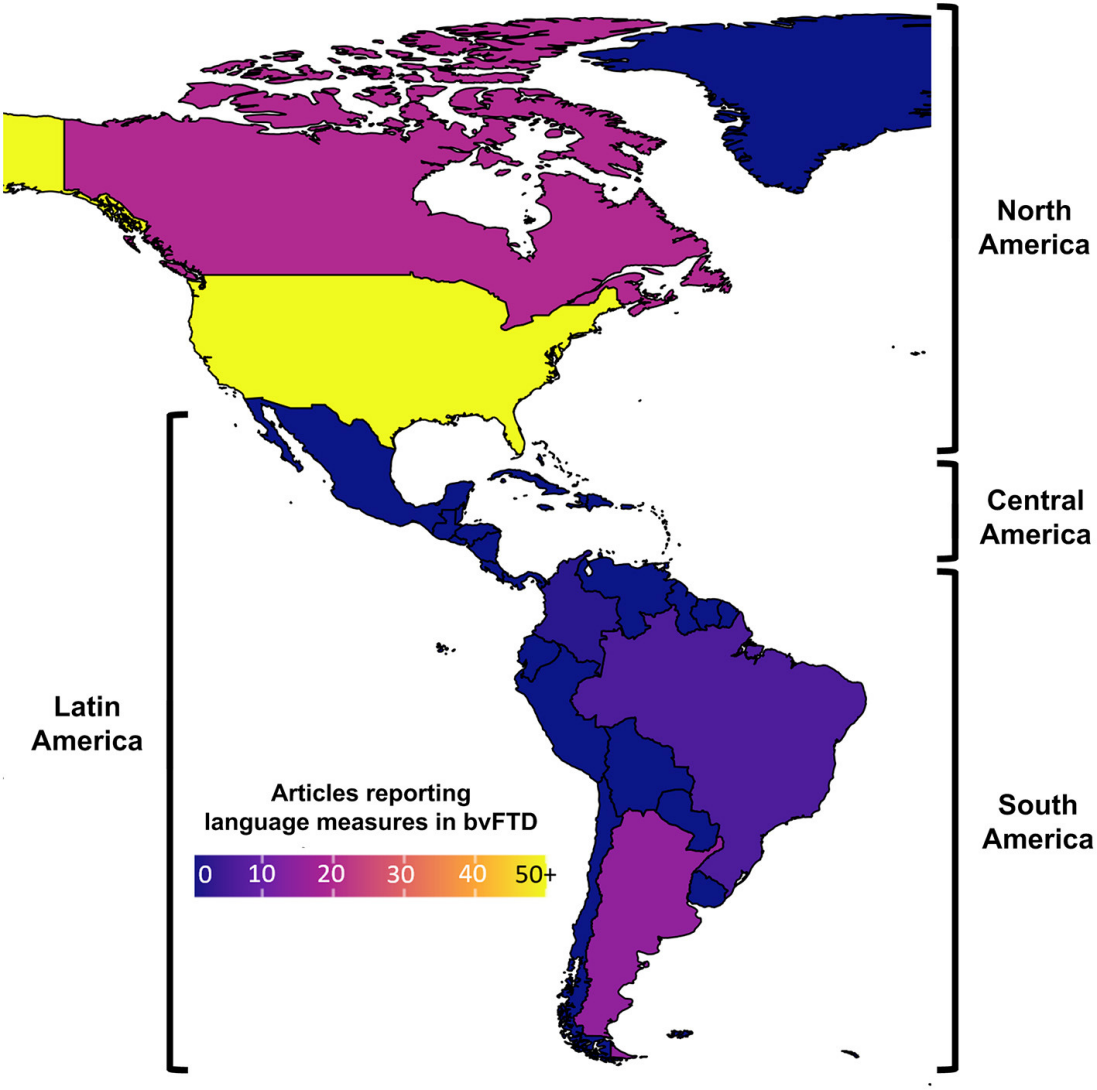
Articles reporting language measures in behavioral variant frontotemporal dementia (bvFTD) cohorts. A systematic review (see Supplementary Material) reveals that, unlike North America (where numerous bvFTD studies have reported language measures), Latin America has produced little evidence on the topic (ranging from low to null, depending on the country).

The latter point may be particularly true in Latin America, where a major increase in the prevalence of bvFTD and other dementias (1, 21, 22) calls for precise clinical phenotyping beyond classical symptoms. Language testing is notoriously scant in regional bvFTD studies. Out of 320 reports that meet inclusion criteria in a systematic review of the topic (23), only 7.5% involve Latin American samples (Figure 1). This hinders valuable opportunities to face mounting regional challenges in the fight against dementia. Indeed, while some gold-standard diagnostic and monitoring methods (e.g., biomarkers) are either limited or broadly unavailable in most local centers (22), linguistic assessments are widely accessible and capture early deficits in bvFTD cohorts across the globe (9) as well as in Latin American individuals with other non-language-dominant disorders, such as Parkinson's and Huntington's disease (24–29).

Moreover, findings from other languages may not generalize to those spoken in Latin America. English, for example, is typified by abundant consonant clusters, genderless nouns, few verb forms, and greater reliance on syntax than prosody for sentential distinctions (30). Conversely, Spanish and Portuguese, the two dominant languages in the region (31), present less frequent consonant clusters, gendered nominal systems, dozens of verb forms, and greater reliance on prosody than syntax to distinguish among sentence types (32). Given that different languages may recruit distinct neural mechanisms (33) and become differently affected by similar brain disruptions (34, 35), novel, language-specific efforts are needed to understand the linguistic profile of Latin American bvFTD (LA bvFTD) patients.

As an initial step, here we contextualize and review language assessments in LA bvFTD cohorts. First, we describe general linguistic features of bvFTD as revealed in research from other world regions. Second, we summarize research conducted in Latin America. Available findings came from fluency, naming, semantic processing, and comprehension tasks. Third, we provide a critical discussion of the evidence and distill its emerging empirical patterns. Finally, we outline key challenges and future directions for the field. This way, we aim to lay the groundwork for a linguistic agenda in LA bvFTD research.

## The General Linguistic Profile of bvFTD

Evidence from other world regions reveals general patterns of affected and spared linguistic functions across bvFTD cohorts, with marked variability for some domains (23). Available results come mainly from studies from North America, Western Europe and Australia, with a marked predominance of English over other languages.

Motor speech is mostly spared (36). Even when they present a strangled-strained voice and articulation difficulties, patients do not exhibit more distortions, false starts, or irregular articulation breakdowns than healthy controls (37). In (semi-) spontaneous tasks, patients may produce shorter segments and abnormal pauses than controls (37). Similar patterns have been documented during text reading (37). However, their production rate is typically normal (38), and so is their rate of phonetic, phonemic, and global speech errors (39).

Performance is also mostly spared in tasks that may be performed through sub-lexical mechanisms. Patients seem unimpaired in phonological manipulation as well as word and sentence repetition (13). Repetition deficits have been observed in only 5% of cases within a large bvFTD cohort (10). On the whole, segmental phonology is widely unaffected in most patients (10, 37). However, patients often exhibit single-word reading (40) and writing (13) deficits.

Conversely, lexical and semantic functions are more systematically impaired in bvFTD. Verbal fluency, across phonemic and semantic conditions, is typically compromised (41, 42). These alterations have been linked to executive deficits (42). As for word retrieval, most studies show picture naming difficulties (43), which may prove more marked for (action) verbs than (object) nouns (13). However, patients seem only sporadically affected when naming faces (44) and smells (45), and they seem unimpaired in sound naming (46). Still, the compromise of semantic abilities appears to be widespread in bvFTD, as deficits have been reported in studies tapping conceptual knowledge (47), word comprehension and definition (48), concept association (38), semantic categorization (49), analogy processing (50), and idiom comprehension (51). Semantic disruptions are also ubiquitous in connected speech. Even though diverse lexical categories are produced with normal frequency (13), patients exhibit more word-finding problems and semantic paraphasias (52). More globally, they have difficulties in accurately reporting events, guiding communication, maintaining global coherence, and organizing discourse (53).

Syntactic processing appears to be preserved in receptive tasks using simple sentences (13). However, impairments are typically observed when using more complex stimuli, such as ambiguous sentences, constructions with synthetic or thematic violations, or discourse-level tasks (51). These difficulties may be secondary to executive deficits (54). Conversely, patients exhibit correct grammar and syntax in (semi)spontaneous production tasks (39).

Briefly, evidence from regions other than Latin American reveals general linguistic patterns in bvFTD patients. Some language domains, such as motor speech and phonology, are partly preserved. Results are more mixed for syntactic skills, with difficulties appearing only during complex tasks. Finally, lexico-semantic abilities, including verbal fluency, appear to be widely impaired. These patterns represent a benchmark for interpreting results from Latin American cohorts, as reviewed next.

## Linguistic Research in LA bvFTD

Following systematic criteria (see Supplementary Materials 1, 2) used in a larger systematic review of language impairments in bvFTD patients (23), we identified 24 papers reporting language assessments in LA bvFTD patients. Beyond one study assessing global language abilities, findings pertain to four main domains: phonemic fluency, semantic fluency, picture naming, and semantic processing (including comprehension). Key findings are described below and detailed in the Table 1. Also, see Supplementary Material 3 for a risk of bias assessment, revealing that only four out of the 24 papers presented high risk of bias.

**Table 1:** Summary of studies reporting language measures in Latin American bvFTD cohorts.

**Author, year**	**Participants**	**Language domain**	**Task**	**Main results**
Lima-Silva et al. (55)	20 bvFTD (mean age: 67.1) 30 AD 34 healthy participants	Global	ACE-R language	bvFTD were impaired when compared to controls but had higher scores than AD patients
Baez et al. (56)	37 bvFTD (mean age: 66) 30 healthy participants	Verbal fluency	Phonological fluency (DKEFS)	A decreased phonological fluency was found in patients
Baez et al. (57)	16 bvFTD (mean age: 65.8) 16 bipolar disorder 22 healthy participants	Verbal fluency	Phonological fluency (“P”)	A decreased phonological fluency was found in patients when compared to control participants but there was no difference when compared to bipolar patients; Lower scores on the phonological fluency test were positively associated with lower GM volumes in the bilateral insula and putamen, the right amygdala, fusiform and inferior frontal gyri, and the left superior temporal gyrus and orbitofrontal cortex
Gleichgerrcht et al. (58)	25 bvFTD (mean age: 70.0) 25 AD 26 healthy participants	Verbal fluency	Phonemic fluency	Phonemic fluency score was lower in bvFTD than in controls but did not differ from AD patients; Phonemic fluency score was correlated to both IFS and FAB scores
Roca et al. (59)	16 high-functioning bvFTD (mean age: 69.1) 19 low-functioning bvFTD (mean age: 65.0) 14 healthy participants	Verbal fluency	Phonemic fluency (FAS)	Phonemic fluency was lower in low-functioning bvFTD when compared to controls and high-functioning bvFTD; these two groups did not differ from each other; these differences were no longer significant when a global mnesic and executive score was introduced as covariate
Russo et al. (60)	27 bvFTD (mean age: 66.5) 46 AD 17 PPA 40 healthy participants	Verbal fluency	Phonemic fluency	Phonemic fluency was lower in bvFTD compared to controls and AD patients patients and did not differ from PPA patients
Santamaria-García et al. (61)	18 bvFTD with apathy (mean age: 58.0) 16 bvFTD with disnhibition (mean age: 57.0)	Verbal fluency	Phonemic fluency	Phonemic fluency scores did not differ between the two bvFTD subgroups
Torralva et al. (62)	20 bvFTD (mean age: 67.2) 10 healthy participants	Verbal fluency	Phonemic fluency (“P”)	Phonemic fluency scores did not differ between the two groups
Torralva et al. (63)	26 mild bvFTD (mean age: 65.8) 14 moderate bvFTD (mean age: 69.9) 18 healthy participants	Verbal fluency	Phonemic fluency (“P”)	Phonemic fluency scores were lower in moderate bvFTD when compared to mild bvFTD and controls and lower in mild bvFTD when compared to controls; Phonemic fluency scores correlated positively with the Faux-Pas scores but not with the Reading Mind in the Eyes scores
Bahia and Viana (64)	12 bvFTD (mean age: 55.9) 12 AD	Verbal fluency	Semantic fluency (animals)	Semantic fluency scores did not differ between bvFTD and AD patients
Boson-Gambogi et al. (65)	29 bvFTD without psychiatric history (mean age: 67.9) 17 bvFTD with psychiatric history (mean age: 65.3)	Verbal fluency	Semantic fluency (animals)	No difference were found between the two groups
Torralva et al. (66)	66 non-vascular bvFTD (mean age: 69.6) 23 vascular bvFTD (mean age: 78.3)	Verbal fluency	Semantic fluency (animals, vegetables)	Non-vascular bvFTD had lower scores for semantic fluency with animals but the scores did not differ for vegetables between the two groups
Wajman et al. (67)	16 bvFTD (mean age: 61.9) 39 AD 22 LBD 48 Amnesic multi-domain MCI 33 Amnesic single-domain MCI 78 healthy participants	Verbal fluency	Semantic fluency (animals)	Semantic fluency scores, number of switches and number and size of clusters did not differ in bvFTD when compared to AD, Amnesic multi-domain MCI and DLB; bvFTD produced less words and less clusters than Amnesic single-domain MCI but did not differ on other measures (cluster size, number of switches); bvFTD produced less words, less and shorter clusters and less switches than controls
Bahia et al. (68)	18 bvFTD (mean age: 70.2) 20 AD 15 healthy participants	Verbal fluency	Phonemic fluency (“P”), semantic fluency (animals)	Both fluency scores were lower in bvFTD than in controls and did not differ between the two patients groups
Couto et al. (69)	22 bvFTD (mean age: 69.8) 10 non-fluent PPA 18 healthy participants	Verbal fluency	Phonemic fluency (“P”), semantic fluency (animals)	Both fluency scores were lower in bvFTD than in controls and did not differ between the two patients groups
Gleichgerrcht et al. (58)	13 bvFTD without dilemma judgment impairment (mean age: 71.4) 9 bvFTD with judgment impairment (mean age: 71.2)	Verbal fluency	Phonemic fluency (“P”), semantic fluency (animals)	Both fluency scores were lower in bvFTD with dilemma judgment impairment than in bvFTD without
Gleichgerrcht et al. (70)	35 bvFTD (mean age: 68.5) 10 PPA 14 healthy participants	Verbal fluency	Phonemic fluency (“P”), semantic fluency (animals)	Both phonemic and semantic fluency scores were lower in bvFTD than in controls; Semantic fluency was lower in PPA than in bvFTD and phonemic fluency did not differ between the two groups
Manes et al. (71)	30 bvFTD with impaired neuropsychological performance (mean age: 69.3) 13 with normal neuropsychological performance (mean age: 67.5) 14 healthy participants	Verbal fluency	Phonemic fluency (“P”), semantic fluency (animals)	Both fluency scores were lower in the neurospychologically impaired bvFTD than in controls but did not differ between non-impaired bvFTD and controls; While phonemic fluency scores were lower in the impaired bvFTD group than in the non-impaired bvFTD group, semantic fluency scores did not differ between the two groups; in the impaired bvFTD subgroup, phonemic and semantic fluency scores correlated with a decision-making task
Mariano et al. (72)	27 bvFTD (mean age: 68.0) 24 AD 25 healthy participants	Verbal fluency	Phonemic fluency (FAS), semantic fluency (animals)	Both fluency scores were lower in bvFTD than in controls and did not differ between the two patients groups
Ramanan et al. (73)	44 bvFTD (mean age: 65.3) 48 AD	Verbal fluency	Phonemic fluency (“A”), semantic fluency	Both fluency scores did not differ between bvFTD and AD patients; phonemic fluency score did not correlate with ToM task score
Reyes et al. (74)	50 bvFTD (mean age: 65.9) 12 nfvPPA 14 svPPA patients 32 healthy participants	Verbal fluency	Phonemic fluency (“P” and “M”), semantic fluency (animals)	Both fluency scores were lower in bvFTD than in controls and higher in bvFTD compared to both nfvPPA and svPPA patients groups
Reyes et al. (75)	26 bvFTD (mean age: 64.4) 20 nfvPPA 20 svPPA patients 33 healthy participants	Verbal fluency	Phonemic fluency, semantic fluency	Both fluency scores were lower in bvFTD than in controls and higher in bvFTD compared to both nfvPPA and svPPA patients groups
Torralva et al. (76)	16 high-ACE bvFTD (mean age: 69.1) 19 low-ACE bvFTD (mean age: 65.0) 10 healthy participants	Verbal fluency	Phonemic fluency (“P”), semantic fluency (animals)	Phonemic fluency was lower in low-ACE bvFTD when compared to controls and high-ACE bvFTD; these two groups did not differ from each other; Phonemic fluency scores did not correlate with a global social cognitive score but did positively correlate with the Reading Mind in the Eyes scores
Couto et al. (69)	22 bvFTD (mean age: 69.8) 10 nfvPPA 18 healthy participants	Naming	Boston Naming Test	Picture naming was impaired in bvFTD patients as well as in the non-fluent PPA patients. bvFTD performance was better than non-fluent PPA.
Gleichgerrcht et al. (58)	13 bvFTD without dilemma judgment impairment (mean age: 71.4) 9 bvFTD with judgment impairment (mean age: 71.2)	Naming	Boston Naming Test	No difference were found between the two groups.
Gleichgerrcht et al. (70)	35 bvFTD (mean age: 68.5) 10 PPA 14 healthy participants	Naming	Boston Naming Test	Picture naming was preserved in bvFTD patients. bvFTD patients presented higher scores than PPA patients.
Manes et al. (71)	30 bvFTD with impaired neuropsychological performance (mean age: 69.3) 13 with normal neuropsychological performance (mean age: 67.5) 14 healthy participants	Naming	Boston Naming Test	Picture naming scores were lower in the neuropychologically impaired bvFTD than in controls but did not differ between neuropsychologically non-impaired bvFTD and controls. The neuropsychologically impaired bvFTD group scores were lower than the non-impaired bvFTD group.
Reyes et al. (75)	26 bvFTD (mean age: 64.4) 20 nfvPPA 20 svPPA patients 33 healthy participants	Naming	Confrontation naming test from Montanes et al. (77)	Picture naming was preserved in vbFTD patients compared to controls. bvFTD patients presented higher scores for both nfvPPA and svPPA.
Roca et al. (59)	16 high-functioning bvFTD (mean age: 69.1) 19 low-functioning bvFTD (mean age: 65.0) 14 healthy participants	Naming	Boston Naming Test	Low-functioning bvFTD differed from both high-functioning bvFTD and healthy controls groups. High-functioning bvFTD patients did not differ from healthy controls.
Russo et al. (60)	27 bvFTD (mean age: 66.5) 46 AD 17 PPA 40 healthy participants	Naming	Boston Naming Test	All patients' groups differed from healthy controls. The bvFTD's group did not differ with the remaining patient's groups.
Santamaria-García et al. (78)	20 bvFTD (mean age: 58.9) 24 AD 20 healthy participants	Naming	Picture-naming task from Snodgrass and Feenan (79)	No significant differences between groups (groups matched by picture naming scores).
Torralva et al. (62)	20 bvFTD with early/mild stage (mean age: 67.2) 10 healthy participants	Naming	Boston Naming Test	Picture naming was impaired in bvFTD patients.
Torralva et al. (76)	16 high-ACE bvFTD (mean age: 69.1) 19 low-ACE bvFTD (mean age: 65.0) 10 healthy participants	Naming	Boston Naming Test	Low-ACE bvFTD differed from both high-ACE bvFTD and healthy controls groups. High-ACE bvFTD patients also differed from healthy controls.
Torralva et al. (63)	66 bvFTD without vascular event history (mean age: 69.6) 23 bvFTD with vascular event history (mean age: 78.3)	Naming	Boston Naming Test	No significant differences between groups.
Gleichgerrcht et al. (70)	35 bvFTD (mean age: 68.5) 10 PPA patients 14 healthy participants	Semantic association	Pyramids and Palm trees	Semantic association was impaired in bvFTD patients compared to controls. BvFTD patients did not differ from PPA patients.
Roca et al. (59)	16 high-functioning bvFTD (mean age: 69.1) 19 low-functioning bvFTD (mean age: 65.0) 14 healthy participants	Semantic association	Pyramids and Palm trees	Low-functioning bvFTD differed from healthy controls. The high-functioning bvFTD group did not differ from both low-functioning and healthy controls groups.
Torralva et al. (62)	20 bvFTD with early/mild stage (mean age: 67.2) 10 healthy controls	Semantic association	Pyramids and Palm trees	Semantic association was impaired in bvFTD patients.
Gleichgerrcht et al. (58)	13 bvFTD without dilemma judgment impairment (mean age: 71.4) 9 bvFTD with judgment impairment (mean age: 71.2)	Comprehension	Token Test	No difference were found between the two groups.
Gleichgerrcht et al. (70)	35 bvFTD (mean age: 68.5) 10 PPA patients 14 healthy participants	Comprehension	Token Test	Comprehension was preserved among all patients groups.
Torralva et al. (62)	20 bvFTD with early/mild stage (mean age: 67.2) 10 healthy controls	Comprehension	Token Test	Comprehension was preserved in bvFTD patients.
Torralva et al. (76)	16 high-ACE bvFTD (mean age: 69.1) 19 low-ACE bvFTD (mean age: 65.0) 10 healthy participants	Comprehension	Token Test	Low-ACE bvFTD differed from both high-ACE bvFTD and healthy controls groups. High-ACE bvFTD patients did not differ from healthy controls.
Reyes et al. (75)	26 bvFTD (mean age: 64.4) 20 nfvPPA (mean age: 63.6) 20 svPPA patients (mean age: 60.3) 33 healthy participants	Comprehension	Proverbs	Proverbs comprehension was impaired in the bvFTD group compared to healthy participants. Moreover, bvFTD also showed better performance than the svPPA group.
Reyes et al. (74)	50 bvFTD (mean age: 65.9) 12 nfvPPA (mean age: 63.63) 14 svPPA patients (mean age: 60.3) 32 healthy participants	Comprehension	Proverbs	All patients' groups differed from healthy controls. The bvFTD group did not differ with the remaining patient groups.

*bvFTD, behavioral variant frontotemporal dementia; AD, Alzheimer's disease; PPA, primary progressive aphasia; svPPA, semantic variant PPA; nfvPPA, non-fluent variant PPA; lvPPA, logopenic variant PPA; ACE, Addenbrooke's Cognitive Assessment*.

### Global Language Skills

One study (55) assessed global language abilities in LA bvFTD patients *via* the ACE-R language subscale, which includes measures of naming, comprehension, repetition, reading, and writing. Results revealed a significant impairment for patients relative to controls. Of note, deficits in the bvFTD cohorts were not milder than those observed in Alzheimer's disease (AD) patients.

### Phonemic Fluency

LA bvFTD patients have impaired phonemic fluency relative to healthy controls (56–58, 60, 68–70, 72, 74, 75). This has been observed for both Spanish-speaking (57, 60, 74, 75) and Portuguese-speaking (68, 72) cohorts, across different age groups (mean age varying from 64.4 to 70.2 years old) and education levels (years of education ranging from 10.8 to 16.0 years). Non-significant differences were reported by Torralva et al. (62), although these results came from a smaller sample with higher MMSE scores than those reported in other studies. Also, phonemic fluency outcomes do not differ significantly between bvFTD and AD [(58, 68, 72, 73), but see (60)]. Comparisons with PPA have yielded mixed results: while some studies report better performance for bvFTD than non-fluent variant PPA and semantic variant PPA patients (74, 75), other found no significant difference between groups (60, 69, 70).^1^ Phonemic fluency performance in LA bvFTD patients has been shown to correlate with the volume of core affected regions –e.g., the bilateral insula and putamen, the right amygdala, fusiform and inferior frontal gyri, and the left superior temporal and orbitofrontal cortices (57).

These impairments may be linked to overall cognitive functioning. LA bvFTD patients with global cognitive difficulties are outperformed by both healthy controls and cognitively preserved LA bvFTD patients (59, 63, 71, 76), there being no difference between the latter two groups [(59, 71, 76), but see (63)]. Phonemic fluency may also be associated with executive (59, 80) and mnesic (59) skills.

The links between this domain and social cognitive functioning are less clear. Phonemic fluency does not seem to be associated with measures of theory of mind (73, 76), empathy (56), or global socio-cognitive skills (76). Also, no difference has been reported in phonemic fluency scores between patients with utilitarian and non-utilitarian moral profiles (80). Note that, beyond social cognition domains, similar phonemic fluency outcomes have been reported between apathetic and disinhibited patients (61). However, positive correlations have been reported between phonemic fluency scores and the Reading-the-Mind-in-the-Eyes test, a Faux-Pas task (63), and a decision-making task (71).

In short, phonemic fluency appears to be compromised in LA bvFTD patients. The severity of this impairment resembles that observed in AD and may even reach the degree of impairment seen in non-fluent and semantic PPA. Reported deficits seem driven by wider executive impairment, whereas their relationship to social cognitive functioning remains poorly understood.

### Semantic Fluency

Semantic fluency assessments also reveal systematic deficits in LA bvFTD samples (58, 67–70, 72, 74, 75). As is the case with phonemic fluency, this impairment is consistent for both Spanish (74, 75) and Portuguese (67, 68, 72), in cohorts with different mean ages (varying from 61.9 to 70.2 years old) and education levels (year of education ranging from 8.7 to 16.0 years). In particular, emerging evidence (67) suggests that, compared with healthy controls, LA bvFTD patients produce fewer and smaller semantic clusters (words retrieved according to semantic subcategories such as pets, birds, or felines, for animals) as well as fewer switches (shifts from one semantic subcategory to another). Semantic fluency deficits in LA bvFTD patients seem less strong than those observed in non-fluent and semantic variant PPA [(70, 74, 75), but see (69)] but as severe as those of patients with amnestic mild cognitive impairment (67) and AD (64, 67, 68, 72, 73).

Such difficulties may be related to global cognitive alterations. Indeed, sub-group analyses reveal that deficits are present in cognitively compromised, but not in cognitive spared, LA bvFTD patients (71, 76). In a similar vein, Wajman et al. (67) found significant positive correlations between semantic fluency measures and MMSE scores.

Additional evidence suggests a link with social cognition skills. Although semantic fluency scores may not differ between patients with utilitarian and non-utilitarian moral profiles (80), they are correlated with decision-making scores (71). Semantic fluency in LA bvFTD cohorts may also be influenced by cerebrovascular disease, as patients without such comorbidity had lower scores on specific categories (animals) (66). Finally, there seems to be no difference in semantic fluency between bvFTD patients with and without psychiatric history (65).

In sum, semantic fluency is systematically impaired in LA bvFTD patients. Deficits are less marked than in PPA variants, but they prove comparable to those of persons with mild cognitive impairment or AD. Such difficulties seem related to more global cognitive and socio-cognitive deficits.

### Picture Naming

Picture naming appears to be mostly impaired in LA bvFTD samples. Available evidence comes from Spanish speakers aged between 65 and 70, with a range of roughly 12–15 years of education. Most studies employed the Boston Naming Test, revealing significant differences between patients and controls (60, 70, 71, 74–76); but see (70). Interestingly, no significant deficits were revealed *via* an experimental naming test designed for AD (75). Moreover, separate studies reported that naming performance in LA bvFTD patients was better than in non-fluent variant and semantic variant PPA (75) and heterogeneous PPA cohorts (70).

Naming deficits might be related to the patients' global cognitive impairment levels, as they prove significantly greater in low- vs. high-functioning LA bvFTD cohorts (59, 71, 76). Indeed, normal naming performance has been reported in the latter subgroup (59). Conversely, picture naming did not differ between patients with utilitarian and non-utilitarian moral profiles (80) or prior history of stroke or silent brain infarcts (66).

Briefly, picture naming seems compromised in LA bvFTD patients, though not as markedly as in PPA variants. These deficits might be driven by the patients' cognitive status, but they seem uninfluenced by socio-cognitive abilities or neurovascular events.

### Semantic Processing and Comprehension

Concept association, as tapped with the Pyramids and Palm Trees test, seems to be impaired in LA bvFTD cohorts (59, 62, 70). However, this pattern seems driven by cognitively impaired patients. In fact, these are outperformed by high-functioning ones, who actually reach normal scores (62). Patients also exhibit deficits in proverb comprehension (74, 75), suggesting impaired figurative language skills. Still, these difficulties are significantly less marked than those of semantic variant PPA and non-fluent variant PPA patients (75).

Conversely, comprehension of increasingly complex commands, as captured by the Token Test, seems globally preserved in LA bvFTD individuals (62, 70). However, this domain also seems sensitive to cognitive decline, as poorer performance has been observed in low- relative to high-functioning patients (76). Furthermore, this domain does not seem to differ between patients with utilitarian and non-utilitarian moral profiles (80).

In sum, LA bvFTD patients seem to exhibit concept association and figurative language comprehension deficits, with preserved abilities to grasp verbal commands. At least some of these patterns might be driven by overall cognitive skills.

## Discussion

Though moderate in quantity and scope, existing findings allow the identification of potential empirical patterns. First, LA bvFTD cohorts exhibit systematic deficits in phonemic and semantic fluency. This impairment is consistent across education levels, age ranges, and in the two languages most widely spoken by Latin Americans: Spanish and Portuguese (31). Interestingly, fluency is also the most consistently disrupted domain across bvFTD patients from other regions, yielding deficits in 76% of cases (10). The detection of naming deficits also aligns with reports showing their presence in more than half of patients (10), matching the incidence of hyperorality, a core diagnostic symptom (3). Difficulties have also been observed in tasks requiring semantic processing and comprehension of complex commands, probably driven by global cognitive deficits.

Despite the widespread dismissal of language deficits in bvFTD, such patterns are not fully surprising. Indeed, the above domains have all been linked to brain regions canonically disrupted in bvFTD. This is true of phonemic fluency, subserved by inferior frontal, insular, and medial temporal regions (81); semantic fluency, linked to frontal, posterior temporal, and inferior parietal regions (81); naming, associated with middle temporal, angular, dorsolateral prefrontal, and inferior frontal regions (82, 83); and semantic processing, underpinned by temporal, inferior/medial prefrontal, occipital, and subcortical regions (84). Compatibly, limited evidence in our review shows that phonemic fluency deficits in Spanish-speaking bvFTD patients are associated with atrophy in inferior frontal, orbitofrontal, and anterior, superior and mesial temporal regions (57). Such links reinforce the relevance of language deficits in the disease.

Comparisons with other diseases illuminate the severity of these impairments in LA bvFTD patients. Deficits in semantic fluency (60, 69, 70), naming (70, 75), semantic association, and comprehension (75) are milder than in PPA variants, which are mainly typified by language impairments (85). One study reported comparable semantic fluency difficulties in LA bvFTD and non-fluent PPA patients (69), potentially driven by partly similar atrophy patterns along frontal regions. Phonemic fluency, which hinges on both linguistic and executive control mechanisms, more consistently yielded similar deficits in LA bvFTD and non-fluent PPA (60, 69, 70), which is mainly distinguished by disruption of language-sensitive fronto-insular networks (85). The latter point could suggest that impaired performance in each syndrome might be driven by different factors, such as executive dysfunction in LA bvFTD and linguistic impairment in PPA (39).

More interestingly, several domains seem as markedly impaired in bvFTD as in AD, a disease in which specific verbal dysfunctions range from frequent (in amnestic presentations) to systematic (in linguistic presentations) (86). In our review, comparable outcomes between these diseases have been reported for global language skills, as evaluated with the ACE-R language scale (55), as well as phonemic (58, 68, 72, 73) and semantic (64, 67, 68, 72, 73) fluency tasks. The same pattern has been reported among speakers of English (87) and Italian (88). However, other domains recruiting both linguistic and executive mechanisms, such as picture naming and syntax, may be differentially affected in LA bvFTD and AD (13, 89), calling for further research on cross-nosological and disease-specific markers.

More generally, evidence from Latin America aligns with global findings supporting the relevance of linguistic assessments in bvFTD, even if these are not primarily affected in the disease (9). In the same vein, previous research has emphasized the usefulness of social cognition assessments in PPA variants, although these syndromes are characterized primarily by language deficits (90). Such approaches underscore the clinical value of assessments that go beyond core symptoms, leading to more exhaustive characterizations to establish individual profiles and personalized plans to treat each patient's more salient disruptions. At the same time, they align with transnosological and dimensional perspectives that frame cognitive outcomes in a continuum between normal and pathological extremes cutting across diseases with different core symptomatology (4). Even deficits that escape core diagnostic criteria may be informative for clinical purposes.

## Challenges and Future Directions

### Gaps in the Study of Language in LA bvFTD Patients

The study of language impairments in bvFTD across Latin America is already informative and promising. However, it is marked by important gaps, especially when compared to work conducted elsewhere. First, the evidence is scant and it secondarily covers only a few, coarse-grained domains, whereas research in other world regions proves more abundant, varied, and granular. In addition, few studies have examined associations between linguistic outcomes, non-verbal cognitive skills, and neural correlates, while none has employed longitudinal designs to evaluate language impairment progression. This hinders the detection of robust and clinically useful patterns, as well as the integration of local results with global findings. The scenario is further complicated by the overlap of patients across reports from the same groups, a problem that also challenges interpretability of findings in other parts of the world.

Second, despite the vast extension of the territory, available results come from only a few centers distributed in three countries (Argentina, Brazil, and Colombia). Accordingly, existing findings may fail to represent the diversity of Latin Americans across regional subgroups–a factor known to affect other aspects of dementia presentation (91). More extensive recruitment across regional clinics and hospitals would be critical to extend the cross-national scope of the evidence. Finally, available data comes only from Portuguese- and Spanish-speaking cohorts, which falls short of capturing the region's linguistic diversity, with over 450 languages (31) and an even larger number of dialects (92). Note that different languages (34), and even different dialects of the same language (93, 94), may become differentially affected by brain disease, so that existing results may not be readily extrapolated across the territory.

Future work should strongly aim to cover these gaps, mainly by acknowledging diversity as a pressing matter and encouraging the exploration of culture-specific variables in a cross-regional agenda. This could be achieved through multicentric efforts, such as those spearheaded by the Consortium to Expand Dementia Research in Latin America–ReDLat (95), offering adequate sample sizes, socio-cultural and dialectal diversity, and ecologically valid measures. In fact, ReDLat is already poised to implement classical (e.g., picture naming) and cutting-edge (e.g., automated speech analyses) tools capturing linguistic features in over 1,000 LA bvFTD patients spanning six countries, two languages (Spanish and Portuguese), and numerous dialects. Moreover, the consortium's multicentric structure is already being leveraged to launch language-focused projects, including novel assessments in bvFTD and AD samples through a combination of automated (acoustic and textual) measures, gold-standard multi-level tests, and validated language profile questionnaires. In the near future, the cross-dialectal scope of these efforts could be fruitfully extended beyond the region through direct contrasts between bvFTD cohorts from Latin America, Spain, and Portugal. This would also cater for a more balanced representation of sites from different countries, as language measures, so far, have been reported in only three bvFTD studies from Spain (96–98) and one from Portugal (99).

Furthermore, these limitations also apply to several other world regions where language studies in bvFTD range from incipient to fully absent. This is the case, for instance, with African countries, most Asian countries, and Russia. Therefore, from a more global perspective, our present call for further Latin American research on the topic should be seen as an instantiation of a broader, cross-national need to be met by the field.

### Clinical and Research Recommendations

This review also highlights the need for Latin American researchers and clinicians to use more sensitive and specific language measures. One of the most systematically assessed domains in LA bvFTD patients is verbal fluency. Although highly useful to detect cognitive impairment in this population, fluency tests are not sufficient to investigate language functioning in bvFTD, calling for more specific tasks.

The Boston Naming Test was the most frequently used naming task in the reviewed studies. However, this test can underestimate Spanish proficiency (100). In this sense, the Multilingual Naming Test might be more culturally and linguistically appropriate to investigate naming abilities in monolingual and multilingual Spanish speakers, and it has been shown to be useful clinically in neurodegenerative populations (101).

The Pyramids and Palm Trees Test was the most frequently used semantic task in our review. As semantic memory is one of the most culturally specific cognitive domains, researchers have developed and validated a culturally and linguistically appropriate version for Spanish speakers, the Pyramids and Pharaohs Test (102). In addition to being shorter (20 vs. 52 trials), this new version also shows a higher sensitivity and specificity to semantic impairments in a Spanish-speaking population.

Finally, the Token Test, which was used frequently in primary studies in the present review, appears appropriate for Latin American patients and it has Spanish and Portuguese norms (103, 104). Nonetheless, no study has investigated motor speech, phonology or syntax in LA bvFTD patients. Prosodic and discourse-based measures, which have also shown to be extremely useful to characterize language impairments in bvFTD patients, have not been used either. Besides a few general language instruments, such as the Bilingual Aphasia Test (105), the Communicative Abilities in Daily Living battery (106), and the Boston Diagnostic Aphasia Examination (107), there is a dearth of fine-grained tools for assessing language in Latin American individuals. The development of such instruments could stimulate regional research on bvFTD and other neurodegenerative conditions.

Moreover, major strides could be made by incorporating automated speech analysis tools (108, 109), which allow capturing multiple acoustic (e.g., prosodic, articulatory) and linguistic (e.g., lexico-semantic, morphosyntactic) features from brief excerpts of natural speech. Relative to standard assessments, this approach presents numerous advantages (e.g., low cost, objective results, ecological validity, scalability), and it has already proven sensitive to bvFTD patients from other world regions (110). In line with recent works on Latin American patients with other neurodegenerative disorders (25, 26), automated speech assessments could open new vistas for translational research on regional bvFTD cohorts.

## Conclusion

The prominence of behavioral and personality changes in bvFTD may have led to a partial dismissal of other cognitive deficits, including linguistic ones. This is unfortunate for underserved regions, such as Latin America, given that language assessments in bvFTD may be sensitive, discriminative, less costly, and more scalable than other diagnostic and monitoring methods. Our review indicates that deficits in verbal fluency, naming, and semantic domains are common and informative across LA bvFTD cohorts, but it also highlights the paucity of evidence, the lack of studies employing fine-grained and cutting-edge tools, and the poor coverage of languages and dialects across the region. Looking forward, multicentric approaches to language in LA bvFTD samples could be of great clinical value, paving the way for more thorough characterizations of patient profiles and novel avenues to support mainstream diagnostic tests.

## Author Contributions

AMG developed the study concept and the study design. AG and MM performed the literature review. AG, MD, MM, and AMG interpreted the data and wrote the manuscript. All authors contributed to the article and approved the submitted version.

## Conflict of Interest

The authors declare that the research was conducted in the absence of any commercial or financial relationships that could be construed as a potential conflict of interest.

## Publisher's Note

All claims expressed in this article are solely those of the authors and do not necessarily represent those of their affiliated organizations, or those of the publisher, the editors and the reviewers. Any product that may be evaluated in this article, or claim that may be made by its manufacturer, is not guaranteed or endorsed by the publisher.

## Footnotes

^1^For comparisons of other fluency measures in connected speech, see Reyes (75).

## References

[1] Custodio N Wheelock A Thumala D Slachevsky A. Dementia in Latin America: epidemiological evidence and implications for public policy. Front Aging Neurosci. (2017) 9:221. 10.3389/fnagi.2017.00221 28751861 PMC5508025

[2] Piguet O Hornberger M Mioshi E Hodges JR. Behavioural-variant frontotemporal dementia: diagnosis, clinical staging, and management. Lancet Neurol. (2011) 10:162-72. 10.1016/S1474-4422(10)70299-4 21147039

[3] Rascovsky K Hodges JR Knopman D Mendez MF Kramer JH Neuhaus J. Sensitivity of revised diagnostic criteria for the behavioural variant of frontotemporal dementia. Brain J Neurol. (2011) 134:2456-77. 10.1093/brain/awr179 PMC3170532 21810890

[4] Ibáñez A García AM Esteves S Yoris A Muñoz E Reynaldo L. Social neuroscience: undoing the schism between neurology and psychiatry. Soc Neurosci. (2018) 13:1–39. 10.1080/17470919.2016.1245214 27707008 PMC11177280

[5] Lanata SC Miller BL. The behavioural variant frontotemporal dementia (bvFTD) syndrome in psychiatry. J Neurol Neurosurg Psychiatry. (2016) 87:501-11. 10.1136/jnnp-2015-310697 PMC4755931 26216940

[6] Bott NT Radke A Stephens ML Kramer JH. Frontotemporal dementia: Diagnosis, deficits and management. Neurodegen Dis Manag. (2014) 4:439-54. 10.2217/nmt.14.34 PMC4824317 25531687

[7] Harciarek M Cosentino S. Language, executive function and social cognition in the diagnosis of frontotemporal dementia syndromes. Int Rev Psychiatry (Abingdon, England). (2013) 25:178-96. 10.3109/09540261.2013.763340 PMC4481322 23611348

[8] Piguet O Hodges JR. Behavioural-variant frontotemporal dementia: an update. Dementia Neuropsychol. (2013) 7:10-8. 10.1590/S1980-57642013DN70100003 PMC5619539 29213814

[9] Garcia AM DeLeon J Tee BL. Neurodegenerative disorders of speech and language: non-language-dominant disease. In*: Reference Module in Neuroscience and Biobehavioral Psychology*. Amsterdam: Elsevier (2020).

[10] Saxon JA Thompson JC Jones M Harris JM Richardson AM Langheinrich T. Examining the language and behavioural profile in FTD and ALS-FTD. J Neurol Neurosurg Psychiatry. (2017) 88:675-80. 10.1136/jnnp-2017-315667 PMC5537548 28596248

[11] Harris JM Jones M Gall C Richardson AMT Neary D du Plessis D. Co-occurrence of language and behavioural change in frontotemporal lobar degeneration. Dementia Geriatr Cogn Disord Extra. (2016) 6:205-13. 10.1159/000444848 PMC4913762 27350781

[12] Cheran G Wu L Lee S Manoochehri M Cines S Fallon E. Cognitive indicators of preclinical behavioral variant frontotemporal dementia in MAPT carriers. J Int Neuropsychol Soc JINS. (2019) 25:184-94. 10.1017/S1355617718001005 PMC6374161 30458895

[13] Hardy CJD Buckley AH Downey LE Lehmann M Zimmerer VC Varley RA. The language profile of behavioral variant frontotemporal dementia. J Alzheimers Dis. (2016) 50:359-71. 10.3233/JAD-150806 PMC4740928 26682693

[14] Warren JD Rohrer JD Rossor MN. Clinical review. Frontotemporal dementia. BMJ (Clinical Research Ed.). (2013) 347:f4827. 10.1136/bmj.f4827 PMC3735339 23920254

[15] Blair M Marczinski CA Davis-Faroque N Kertesz A. A longitudinal study of language decline in Alzheimer's disease frontotemporal dementia. J Int Neuropsychol Soc. (2007) 13:237-45. 10.1017/S1355617707070269 17286881

[16] Seeley WW. Frontotemporal dementia neuroimaging: a guide for clinicians. Front Neurol Neurosci. (2009) 24:160-7. 10.1159/000197895 19182474

[17] Ardila A Bernal B Rosselli M. How localized are language brain areas? A review of brodmann areas involvement in oral language. Arch Clin Neuropsychol. (2016) 31:112-22. 10.1093/arclin/acv081 26663825

[18] Hagoort P. The neurobiology of language beyond single-word processing. Science (New York, N.Y.). (2019) 366:55-8. 10.1126/science.aax0289 31604301

[19] Hickok G. The functional neuroanatomy of language. Phys Life Rev. (2009) 6:121-43. 10.1016/j.plrev.2009.06.001 PMC2747108 20161054

[20] Oh A Duerden EG Pang EW. The role of the insula in speech and language processing. Brain Lang. (2014) 135:96-103. 10.1016/j.bandl.2014.06.003 PMC4885738 25016092

[21] GBD 2016 Dementia Collaborators. Global, regional, and national burden of Alzheimer's disease and other dementias, 1990-2016: a systematic analysis for the Global Burden of Disease Study 2016. Lancet Neurol. (2019) 18:88-106. 10.1016/S1474-4422(18)30403-4 PMC6291454 30497964

[22] Parra MA Baez S Sedeño L Gonzalez Campo C Santamaría-García H Aprahamian I. Dementia in Latin America: paving the way toward a regional action plan. Alzheimers Dementia J Alzheimers Assoc. (2021) 17:295-313. 10.1002/alz.12202 PMC7984223 33634602

[23] Geraudie A Battista P García AM Allen IE Miller ZA Gorno-Tempini ML. Speech and language impairments in behavioral variant frontotemporal dementia: a systematic review. MedRxiv. (2020). 10.1101/2021.07.10.21260313 PMC12169595 34673112

[24] Birba A García-Cordero I Kozono G Legaz A Ibáñez A Sedeño L. Losing ground: Frontostriatal atrophy disrupts language embodiment in Parkinson's and Huntington's disease. Neurosci Biobehav Rev. (2017) 80:673-87. 10.1016/j.neubiorev.2017.07.011 28780312

[25] Eyigoz E Courson M Sedeño L Rogg K Orozco-Arroyave JR Nöth E. From discourse to pathology: automatic identification of Parkinson's disease patients *via* morphological measures across three languages. Cortex J Devoted Study Nervous Syst Behav. (2020) 132:191-205. 10.1016/j.cortex.2020.08.020 PMC7655620 32992069

[26] García AM Carrillo F Orozco-Arroyave JR Trujillo N Vargas Bonilla JF Fittipaldi S. How language flows when movements don't: an automated analysis of spontaneous discourse in Parkinson's disease. Brain Lang. (2016) 162:19-28. 10.1016/j.bandl.2016.07.008 27501386

[27] García AM Sedeño L Trujillo N Bocanegra Y Gomez D Pineda D. Language deficits as a preclinical window into parkinson's disease: evidence from asymptomatic parkin dardarin mutation carriers. J Int Neuropsychol Soc. (2017) 23:150-8. 10.1017/S1355617716000710 28205494

[28] García AM Bocanegra Y Herrera E Moreno L Carmona J Baena A. Parkinson's disease compromises the appraisal of action meanings evoked by naturalistic texts. Cortex J Devoted Study Nerv Syst Behav. (2018) 100:111-26. 10.1016/j.cortex.2017.07.003 28764852

[29] Salmazo-Silva H Parente MA de MP Rocha MS Baradel RR Cravo AM. Lexical-retrieval and semantic memory in Parkinson's disease: The question of noun and verb dissociation. Brain Lang. (2017) 165:10-20. 10.1016/j.bandl.2016.10.006 27912072

[30] Halliday MAK Matthiessen C Halliday M. An Introduction to Functional Grammar. Birmingham: Routledge (2014). 10.4324/9780203783771

[31] Eberhard D Simons GF. Ethnologue: Languages of the World. 23rd ed. Dallas: Sil International, Global Publishing (2020).

[32] Wetzels WL Menuzzi S Costa J. The Handbook of Portuguese Linguistics Hoboken, NJ: Wiley (2020).

[33] Evans N Levinson SC. The myth of language universals: language diversity and its importance for cognitive science. Behav Brain Sci. (2009) 32:429-48; discussion 448–494. 10.1017/S0140525X0999094X 19857320

[34] Canu E Agosta F Battistella G Spinelli EG DeLeon J Welch AE. Speech production differences in English and Italian speakers with nonfluent variant PPA. Neurology. (2020) 94:e1062-72. 10.1212/WNL.0000000000008879 PMC7238919 31924679

[35] Paradis M. The need for awareness of aphasia symptoms in different languages. J Neurolinguist. (2001) 14:85-91. 10.1016/S0911-6044(01)00009-4

[36] Deleon J Gesierich B Besbris M Ogar J Henry ML Miller BL. Elicitation of specific syntactic structures in primary progressive aphasia. Brain Lang. (2012) 123:183-90. 10.1016/j.bandl.2012.09.004 PMC3502680 23046707

[37] Vogel AP Poole ML Pemberton H Caverlé MWJ Boonstra FMC Low E. Motor speech signature of behavioral variant frontotemporal dementia: refining the phenotype. Neurology. (2017) 89:837-44. 10.1212/WNL.0000000000004248 28733335

[38] Ash S Evans E O'Shea J Powers J Boller A Weinberg D. Differentiating primary progressive aphasias in a brief sample of connected speech. Neurology. (2013) 81:329-36. 10.1212/WNL.0b013e31829c5d0e PMC3772830 23794681

[39] Ash S Nevler N Phillips J Irwin DJ McMillan CT Rascovsky K. A longitudinal study of speech production in primary progressive aphasia and behavioral variant frontotemporal dementia. Brain Lang. (2019) 194:46-57. 10.1016/j.bandl.2019.04.006 PMC6656376 31075725

[40] Downey LE Mahoney CJ Buckley AH Golden HL Henley SM Schmitz N. White matter tract signatures of impaired social cognition in frontotemporal lobar degeneration. NeuroImage Clin. (2015) 8:640-51. 10.1016/j.nicl.2015.06.005 PMC4513187 26236629

[41] Irwin DJ McMillan CT Xie SX Rascovsky K Van Deerlin VM Coslett HB. Asymmetry of post-mortem neuropathology in behavioural-variant frontotemporal dementia. Brain J Neurol. (2018) 141:288-301. 10.1093/brain/awx319 PMC5837322 29228211

[42] Libon DJ McMillan C Gunawardena D Powers C Massimo L Khan A. Neurocognitive contributions to verbal fluency deficits in frontotemporal lobar degeneration. Neurology. (2009) 73:535-42. 10.1212/WNL.0b013e3181b2a4f5 PMC2730797 19687454

[43] Whitwell JL Przybelski SA Weigand SD Ivnik RJ Vemuri P Gunter JL. Distinct anatomical subtypes of the behavioural variant of frontotemporal dementia: a cluster analysis study. Brain J Neurol. (2009) 132:2932-46. 10.1093/brain/awp232 PMC2768663 19762452

[44] Kamminga J Kumfor F Burrell JR Piguet O Hodges JR Irish M. Differentiating between right-lateralised semantic dementia and behavioural-variant frontotemporal dementia: an examination of clinical characteristics and emotion processing. J Neurol Neurosurg Psychiatry. (2015) 86:1082-8. 10.1136/jnnp-2014-309120 25511791

[45] Luzzi S Snowden JS Neary D Coccia M Provinciali L Lambon Ralph MA. Distinct patterns of olfactory impairment in Alzheimer's disease, semantic dementia, frontotemporal dementia, corticobasal degeneration. Neuropsychologia. (2007) 45:1823-31. 10.1016/j.neuropsychologia.2006.12.008 17270222

[46] Lin P-H Chen H-H Chen N-C Chang W-N Huang C-W Chang Y-T. Anatomical correlates of non-verbal perception in dementia patients. Front Aging Neurosci. (2016) 8:207. 10.3389/fnagi.2016.00207 27630558 PMC5005819

[47] Irish M Eyre N Dermody N O'Callaghan C Hodges JR Hornberger M. Neural substrates of semantic prospection-evidence from the dementias. Front Behav Neurosci. (2016) 10:96. 10.3389/fnbeh.2016.00096 27252632 PMC4877391

[48] Chen Y Kumfor F Landin-Romero R Irish M Hodges JR Piguet O. Cerebellar atrophy and its contribution to cognition in frontotemporal dementias. Ann Neurol. (2018) 84:98-109. 10.1002/ana.25271 30014499

[49] Hughes LE Nestor PJ Hodges JR Rowe JB. Magnetoencephalography of frontotemporal dementia: spatiotemporally localized changes during semantic decisions. Brain A J Neurol. (2011) 134:2513-22. 10.1093/brain/awr196 PMC3170535 21840892

[50] Krawczyk DC Morrison RG Viskontas I Holyoak KJ Chow TW Mendez MF. Distraction during relational reasoning: the role of prefrontal cortex in interference control. Neuropsychologia. (2008) 46:2020-32. 10.1016/j.neuropsychologia.2008.02.001 18355881

[51] Luzzi S Baldinelli S Ranaldi V Fiori C Plutino A Fringuelli FM. The neural bases of discourse semantic and pragmatic deficits in patients with frontotemporal dementia and Alzheimer's disease. Cortex J Devoted Study Nervous Syst Behav. (2020) 128:174-91. 10.1016/j.cortex.2020.03.012 32353756

[52] Wilson SM Henry ML Besbris M Ogar JM Dronkers NF Jarrold W. Connected speech production in three variants of primary progressive aphasia. Brain J Neurol. (2010) 133:2069-88. 10.1093/brain/awq129 PMC2892940 20542982

[53] Healey M Spotorno N Olm C Irwin DJ Grossman M. Cognitive neuroanatomic accounts of referential communication in focal dementia. ENeuro. (2019) 6:1–35. 10.1523/ENEURO.0488-18.2019 PMC6794081 31451606

[54] Peelle JE Cooke A Moore P Vesely L Grossman M. Syntactic and thematic components of sentence processing in progressive nonfluent aphasia and nonaphasic frontotemporal dementia. J Neurolinguist. (2007) 20:482-94. 10.1016/j.jneuroling.2007.04.002 PMC2083702 18978927

[55] Lima-Silva TB Bahia VS Carvalho VA Guimarães HC Caramelli P Balthazar MLF. Direct and indirect assessments of activities of daily living in behavioral variant frontotemporal dementia and Alzheimer disease. J Geriatr Psychiatry Neurol. (2015) 28:19-26. 10.1177/0891988714541874 25015849

[56] Baez S Manes F Huepe D Torralva T Fiorentino N Richter F. Primary empathy deficits in frontotemporal dementia. Front Aging Neurosci. (2014) 6:262. 10.3389/fnagi.2014.00262 25346685 PMC4193328

[57] Baez S Pinasco C Roca M Ferrari J Couto B García-Cordero I. Brain structural correlates of executive and social cognition profiles in behavioral variant frontotemporal dementia and elderly bipolar disorder. Neuropsychologia. (2019) 126:159-69. 10.1016/j.neuropsychologia.2017.02.012 28219620

[58] Gleichgerrcht E Roca M Manes F Torralva T. Comparing the clinical usefulness of the Institute of Cognitive Neurology (INECO) frontal screening (IFS) and the frontal assessment battery (FAB) in frontotemporal dementia. J Clin Exp Neuropsychol. (2011) 33:997-1004. 10.1080/13803395.2011.589375 21923634

[59] Roca M Manes F Gleichgerrcht E Watson P Ibáñez A Thompson R. Intelligence and executive functions in frontotemporal dementia. Neuropsychologia. (2013) 51:725-30. 10.1016/j.neuropsychologia.2013.01.008 PMC3610016 23347963

[60] Russo G Russo MJ Buyatti D Chrem P Bagnati P Fernández Suarez M. Utility of the Spanish version of the FTLD-modified CDR in the diagnosis and staging in frontotemporal lobar degeneration. J Neurol Sci. (2014) 344:63-8. 10.1016/j.jns.2014.06.024 25015844

[61] Santamaría-García H Reyes P García A Baéz S Martinez A Santacruz JM. First symptoms neurocognitive correlates of behavioral variant frontotemporal dementia. J Alzheimers Dis. (2016) 54:957-70. 10.3233/JAD-160501 27567867

[62] Torralva T Kipps CM Hodges JR Clark L Bekinschtein T Roca M. The relationship between affective decision-making and theory of mind in the frontal variant of fronto-temporal dementia. Neuropsychologia. (2007) 45:342-9. 10.1016/j.neuropsychologia.2006.05.031 16893555

[63] Torralva T Gleichgerrcht E Torres Ardila MJ Roca M Manes FF. Differential cognitive and affective theory of mind abilities at mild and moderate stages of behavioral variant frontotemporal dementia. Cogn Behav Neurol. (2015) 28:63-70. 10.1097/WNN.0000000000000053 26102996

[64] Bahia VS Viana R. Accuracy of neuropsychological tests and the Neuropsychiatric Inventory in differential diagnosis between Frontotemporal dementia and Alzheimer's disease. Dementia Neuropsychol. (2009) 3:332–36. 10.1590/S1980-57642009DN30400012 PMC5619421 29213649

[65] Gambogi LB Guimarães HC de Souza LC Caramelli P. Long-term severe mental disorders preceding behavioral variant frontotemporal dementia: frequency clinical correlates in an outpatient sample. J Alzheimers Dis. (2018) 66:1577-85. 10.3233/JAD-180528 30452412

[66] Torralva T Sposato LA Riccio PM Gleichgerrcht E Roca M Toledo JB. Role of brain infarcts in behavioral variant frontotemporal dementia: Clinicopathological characterization in the National Alzheimer's Coordinating Center database. Neurobiol Aging. (2015) 36:2861-8. 10.1016/j.neurobiolaging.2015.06.026 PMC4562890 26220367

[67] Wajman JR Cecchini MA Bertolucci PHF Mansur LL. Quanti-qualitative components of the semantic verbal fluency test in cognitively healthy controls, mild cognitive impairment, dementia subtypes. Appl Neuropsychol Adult. (2019) 26:533-42. 10.1080/23279095.2018.1465426 30375889

[68] Bahia VS Cecchini MA Cassimiro L Viana R Lima-Silva TB de Souza LC. The accuracy of INECO frontal screening in the diagnosis of executive dysfunction in frontotemporal dementia and Alzheimer disease. Alzheimer Dis Assoc Disord. (2018) 32:314-9. 10.1097/WAD.0000000000000255 29734264

[69] Couto B Manes F Montañés P Matallana D Reyes P Velasquez M. Structural neuroimaging of social cognition in progressive non-fluent aphasia and behavioral variant of frontotemporal dementia. Front Hum Neurosci. (2013) 7:467. 10.3389/fnhum.2013.00467 23966929 PMC3744869

[70] Gleichgerrcht E Torralva T Roca M Szenkman D Ibanez A Richly P. Decision making cognition in primary progressive aphasia. Behav Neurol. (2012) 25:45-52. 10.1155/2012/606285 PMC5294273 22207422

[71] Manes F Torralva T Ibáñez A Roca M Bekinschtein T Gleichgerrcht E. Decision-making in frontotemporal dementia: clinical, theoretical and legal implications. Dementia Geriatr Cogn Disord. (2011) 32:11-7. 10.1159/000329912 21822019

[72] Mariano LI O'Callaghan C Guimarães HC Gambogi LB da Silva TBL Yassuda MS. Disinhibition in frontotemporal dementia Alzheimer's disease: a neuropsychological behavioural investigation. J Int Neuropsychol Soc. (2020) 26:163-71. 10.1017/S1355617719000973 31543087

[73] Ramanan S de Souza LC Moreau N Sarazin M Teixeira AL Allen Z. Determinants of theory of mind performance in Alzheimer's disease: a data-mining study. Cortex J Devoted Study Nerv Syst Behav. (2017) 88:8-18. 10.1016/j.cortex.2016.11.014 28012370

[74] Reyes P Ortega-Merchan MP Rueda A Uriza F Santamaria-García H Rojas-Serrano N. Functional connectivity changes in behavioral, semantic, and nonfluent variants of frontotemporal dementia. Behav Neurol. (2018) 2018:9684129. 10.1155/2018/9684129 29808100 PMC5902123

[75] Reyes PA Rueda ADP Uriza F Matallana DL. Networks disrupted in linguistic variants of frontotemporal dementia. Front Neurol. (2019) 10:903. 10.3389/fneur.2019.00903 31507513 PMC6716200

[76] Torralva T Roca M Gleichgerrcht E Bekinschtein T Manes F. A neuropsychological battery to detect specific executive and social cognitive impairments in early frontotemporal dementia. Brain J Neurol. (2009) 132:1299-309. 10.1093/brain/awp041 19336463

[77] Montanes P Goldblum MC Boller F. The naming impairment of living and nonliving items in Alzheimer's disease. J Int Neuropsychol Soc. (1995) 1:39–48. 10.1017/S1355617700000084 9375207

[78] Santamaría-García H Baez S Reyes P Santamaría-García JA Santacruz-Escudero JM Matallana D. (2017). A lesion model of envy and Schadenfreude?: Legal, deservingness and moral dimensions as revealed by neurodegeneration. Brain: A J Neurol., 140, 3357–3377. 10.1093/brain/awx269 PMC5841144 29112719

[79] Snodgrass JG Feenan K. Priming effects in picture fragment completion: support for the perceptual closure hypothesis. J Exp Psychol Gen. (1990) 119:276. 2145392 10.1037//0096-3445.119.3.276

[80] Gleichgerrcht E Torralva T Roca M Pose M Manes F. The role of social cognition in moral judgment in frontotemporal dementia. Soc Neurosci. (2011) 6:113-22. 10.1080/17470919.2010.506751 20706963

[81] Vonk JMJ Rizvi B Lao PJ Budge M Manly JJ Mayeux R. Letter and category fluency performance correlates with distinct patterns of cortical thickness in older adults. Cerebral Cortex (New York, N.Y.: 1991). (2019) 29:2694-700. 10.1093/cercor/bhy138 PMC6519688 29893804

[82] Garn CL Allen MD Larsen JD. An fMRI study of sex differences in brain activation during object naming. Cortex J Devoted Study Nerv Syst Behav. (2009) 45:610-8. 10.1016/j.cortex.2008.02.004 18639870

[83] Price CJ Moore CJ Humphreys GW Frackowiak RS Friston KJ. The neural regions sustaining object recognition and naming. Proc Biol Sci. (1996) 263:1501-7. 10.1098/rspb.1996.0219 8952093

[84] Fang Y Han Z Zhong S Gong G Song L Liu F. The semantic anatomical network: evidence from healthy and brain-damaged patient populations. Hum Brain Map. (2015) 36:3499-515. 10.1002/hbm.22858 PMC6869673 26059098

[85] Gorno-Tempini ML Hillis AE Weintraub S Kertesz A Mendez M Cappa SF. Classification of primary progressive aphasia and its variants. Neurology. (2011) 76:1006-14. 10.1212/WNL.0b013e31821103e6 PMC3059138 21325651

[86] Verma M Howard RJ. Semantic memory and language dysfunction in early Alzheimer's disease: a review. Int J Geriatr Psychiatry. (2012) 27:1209-17. 10.1002/gps.3766 22298328

[87] Hodges JR Patterson K Ward R Garrard P Bak T Perry R. The differentiation of semantic dementia and frontal lobe dementia (temporal and frontal variants of frontotemporal dementia) from early Alzheimer's disease: a comparative neuropsychological study. Neuropsychology. (1999) 13:31-40. 10.1037/0894-4105.13.1.31 10067773

[88] Filippi M Basaia S Canu E Imperiale F Meani A Caso F. Brain network connectivity differs in early-onset neurodegenerative dementia. Neurology. (2017) 89:1764-72. 10.1212/WNL.0000000000004577 PMC5664301 28954876

[89] Hutchinson AD Mathias JL. Neuropsychological deficits in frontotemporal dementia and Alzheimer's disease: a meta-analytic review. J Neurol Neurosurg Psychiatry. (2007) 78:917-28. 10.1136/jnnp.2006.100669 PMC2117891 17371908

[90] Fittipaldi S Ibanez A Baez S Manes F Sedeno L Garcia AM. More than words: social cognition across variants of primary progressive aphasia. Neurosci Biobehav Rev. (2019) 100:263-84. 10.1016/j.neubiorev.2019.02.020 30876954

[91] Parra MA Baez S Allegri R Nitrini R Lopera F Slachevsky A. Dementia in Latin America: assessing the present and envisioning the future. Neurology. (2018) 90:222-31. 10.1212/WNL.0000000000004897 PMC5791795 29305437

[92] Lipski JM. Dialects of Spanish and Portuguese. In: Boberg C, Nerbonne J, Watt D, editors. The Handbook of Dialectology. 1st ed. John Wiley & Sons, Inc (2018).

[93] Fabbro F Paradis M. Differential impairments in four multilingual patients with subcortical lesions. In: Paradis M, editor. Aspects of Bilingual Aphasia. 1st Edn. Bingley, UK: Emerald Group Pub Ltd (1995). p. 139-76.

[94] García AR. Translating with an Injured Brain: Neurolinguistic Aspects of Translation as Revealed by Bilinguals with Cerebral Lesions. Meta: Translators' J. (2015) 60:112–34.

[95] Ibanez A Yokoyama JS Possin KL Matallana D Lopera F Nitrini R. The multi-partner consortium to expand dementia research in Latin America (ReDLat): driving multicentric research and implementation science. Front Neurol. (2021) 12:631722. 10.3389/fneur.2021.631722 33776890 PMC7992978

[96] Fernández-Matarrubia M Matías-Guiu JA Cabrera-Martín MN Moreno-Ramos T Valles-Salgado M Carreras JL. Episodic memory dysfunction in behavioral variant frontotemporal dementia: a clinical and FDG-PET study. J Alzheimers Dis. (2017) 57:1251-64. 10.3233/JAD-160874 28304289

[97] Pozueta A Lage C García-Martínez M Kazimierczak M Bravo M López-García S. Cognitive behavioral profiles of left right semantic dementia: differential diagnosis with behavioral variant frontotemporal dementia Alzheimer's Disease. J Alzheimers Dis. (2019) 72:1129-44. 10.3233/JAD-190877 31683488

[98] Pozueta A Lage C Martínez MG Kazimierczak M Bravo M López-García S. A brief drawing task for the differential diagnosis of semantic dementia. J Alzheimers Dis. (2019) 72:151-60. 10.3233/JAD-190660 31561372

[99] Lemos R Duro D Simões MR Santana I. The free and cued selective reminding test distinguishes frontotemporal dementia from Alzheimer's disease. Arch Clin Neuropsychol. (2014) 29:670-9. 10.1093/arclin/acu031 PMC4263917 25062746

[100] Gollan TH Weissberger GH Runnqvist E Montoya RI Cera CM. Self-ratings of spoken language dominance: a multi-lingual naming test (MINT) and preliminary norms for young and aging Spanish-english Bilinguals. Bilingualism (Cambridge, England). (2012) 15:594-615. 10.1017/S1366728911000332 PMC4212892 25364296

[101] Ivanova I Salmon DP Gollan TH. The multilingual naming test in Alzheimer's disease: clues to the origin of naming impairments. J Int Neuropsychol Soc. (2013) 19:272-83. 10.1017/S1355617712001282 PMC4356120 23298442

[102] Martinez Cuitiño M Barreyro JP. Pyramids and palm trees or pyramids and pharaohs?: adaptation and validation of semantic association test to the spanish. Interdisciplinaria. (2010) 27:247–60.

[103] Fontanari JL. O ≪ token test ≫: Elegância e concisäo na avaliaçäo da compreensäo do afásico. Validaçäo da versäo reduzida de Renzi para o português. Neurobiologia. (1989) 52:177-218.

[104] Peña-Casanova J Quiñones-Úbeda S Gramunt-Fombuena N Aguilar M Casas L Molinuevo JL. Spanish Multicenter Normative Studies (NEURONORMA Project): norms for Boston naming test and token test. Arch Clin Neuropsychol. (2009) 24:343-54. 10.1093/arclin/acp039 19648582

[105] Paradis M Ardila A. Prueba de afasia para bilingües (American Spanish version. Hillsdale, NJ: Lawrence Erlbaum (1989).

[106] Martín P Manning L Muñoz P Montero I. Communicative abilities in daily living: Spanish standardization. Eval Psicol. (1990) 6:369-84.

[107] Pineda DA Rosselli M Ardila A Mejia SE Romero MG Perez C. The boston diagnostic aphasia examination-Spanish version: the influence of demographic variables. J Int Neuropsychol Soc. (2000) 6:802-14. 10.1017/S135561770067707X 11105470

[108] Boschi V Catricalà E Consonni M Chesi C Moro A Cappa SF. Connected speech in neurodegenerative language disorders: a review. Front Psychol. (2017) 8:269. 10.3389/fpsyg.2017.00269 28321196 PMC5337522

[109] de la Fuente Garcia S Ritchie CW Luz S. Artificial intelligence, speech, language processing approaches to monitoring Alzheimer's disease: a systematic review. J Alzheimers Dis. (2020) 78:1547-74. 10.3233/JAD-200888 PMC7836050 33185605

[110] Zimmerer VC Hardy CJD Eastman J Dutta S Varnet L Bond RL. Automated profiling of spontaneous speech in primary progressive aphasia and behavioral-variant frontotemporal dementia: an approach based on usage-frequency. Cortex J Devoted Study Nerv Syst Behav. (2020) 133:103-19. 10.1016/j.cortex.2020.08.027 33120189

