# Supplementary material for: Language in Behavioral Variant Frontotemporal Dementia: Another Stone to Be Turned in Latin America
Source: Front Neurol. 2021 Aug 10;12:702770. doi: 10.3389/fneur.2021.702770 (PMC8383282; doi:10.3389/fneur.2021.702770)
Supplement: Supplementary file 1 [file Data_Sheet_1.docx]

**Supplementary material**

**1. Search strategy**

A systematic review of the literature was conducted on five databases (PubMed, Embase, Web of Science, PsycINFO, and Cochrane CENTRAL) with the following search terms: (“Language disorders” (as a MeSH term), “Aphasia” (as a MeSH term), “Language”, “Linguistic”, “aphasia”, “aphasic”, “speech”, “naming”, “anomic”, “anomia”, “semantic”, “semantics”, “syntax”, “syntactic”, “prosody”, “prosodic”, “phonology”, “phonological”, “discourse”, “picture description”, “fluency”, “grammar”, “agrammatism”, ”agrammatic”, “writing”, “write”, “reading”, “read”, “comprehension”, “repetition”, “dysarthria”, “spell”, “spelling”, “communication”, “word”, “sentence”, “verb”, “verbal”, “noun”, “lexical”, “lexicon”, “dyslexia”, “dyslexic”, “alexia”, “alexic) AND (“bvFTD” or (“behavioral” or “behavioural”) and (“frontotemporal lobar degeneration” (as a MeSH term) or “ftld”)) OR ((“behavioral” or “behavioural”) and (“frontotemporal” or “fronto-temporal”) and (“dementia” or “dementias” or “degeneration”)) OR ((“behavioral” or “behavioural”) and (“ftd”)). Duplicates were removed and only published articles or articles in press were retained.

**2. Study selection**

For this review, articles were selected based on the following criteria: (1) article written in English; (2) experimental study in humans (excluding reviews and animal studies); (3) inclusion of at least 5 bvFTD patients (excluding FTD-ALS patients or general FTD groups); (4) bvFTD diagnosis based on accepted international consensus criteria (Neary et al., 1998 or Rascovsky et al., 2011); (5) presence of a comparison group; and (6) data about performance on at least one language task.

**3. Risk of bias assessment**

The risk of bias in studies was assessed at the study level using the Newcastle Ottawa Scale (NOS) (Wells et al., 2000). For the purpose of this review (Geraudie et al., submitted), we adapted the existing tool and assessed each study for (1) sample representativeness, (2) sample size, (3) comparability between patients and controls, (4) ascertainment of language symptoms, and (5) statistical quality –Supplementary Tables 1 and 2. An overall risk of bias judgement summarizing across items was made. For the interpretation of total scores, which range from 0 to 5, studies were judged to be of low risk of bias (≥ 3 points) or high risk of bias (< 3 points).

**Supplementary Table 1.** Modified Newcastle-Ottawa Scale and scoring guide.*

| **1. Representativeness of the sample (is the case definition adequate?)**  1 point: Requires some independent validation by a multidisciplinary team. The case was defined using multiple sources of information (neurology, neuropsychology, etc.).  0.5 point: No independent validation, but the case was defined using multiple sources of information (neurology, neuropsychology, etc.).  0 point: No description, not done.  **2. Sample size**  1 point: Sample size was greater than 30 participants.  0.5 point: Sample size was between 20 and 30 participants.  0 point: Sample size was less than 20 participants or a convenience sample.  **3. Comparability of cases and controls based on the design or analysis.**  1 point: Either cases and controls must be matched in the design or confounders (i.e., age, sex, education) must be adjusted for in the analysis.  0.5 point: Cases and controls are only partially matched in the design or confounders (i.e., age, sex, education).  0 point: Both cases and control are not matched in the design and confounders are not adjusted for in the analysis.  **4. Ascertainment of language impairment**  1 point: Validated measurement tool.  0.5 point: Both validated and non-validated measurement tool.  0 point: no description.  **5. Quality of descriptive statistics reporting**  1 point: Reported descriptive statistics to describe the population (e.g., language measures) with a proper measure of dispersion (*e.g.*, standard deviation, standard error, effect size, range, distribution on the graph).  0 point: Descriptive statistics were not reported, were incomplete, or did not include proper measures of dispersion. |
| --- |
| * Link: <http://www.ohri.ca/programs/clinical_epidemiology/oxford.asp> |

**Supplementary Table 2.** Risk of bias in the included studies.

| **Author, year** | Representativeness | Size | Comparability | Outcome | Statistics | Total |
| --- | --- | --- | --- | --- | --- | --- |
| Baez et al., 2014 | 1 | 1 | 1 | 1 | 1 | 5 |
| Baez et al., 2019 | 1 | 0 | 1 | 1 | 1 | 4 |
| Bahia et al., 2009 | 0.5 | 0 | 0 | 1 | 1 | 2.5 |
| Bahia et al., 2018 | 0.5 | 0 | 0 | 1 | 1 | 2.5 |
| Boson-Gambogi et al., 2018 | 1 | 1 | 0 | 0 | 0 | 2 |
| Couto et al., 2013 | 1 | 0.5 | 1 | 1 | 1 | 4.5 |
| Gleichgerrcht et al., 2011 | 0.5 | 0.5 | 0.5 | 1 | 1 | 3.5 |
| Gleichgerrcht et al., 2011 | 0.5 | 0.5 | 0 | 1 | 1 | 3 |
| Gleichgerrcht et al., 2012 | 1 | 1 | 1 | 0 | 0 | 3 |
| Lima-Silva et al., 2015 | 0.5 | 0.5 | 1 | 1 | 1 | 4 |
| Manes et al., 2011 | 1 | 1 | 1 | 1 | 1 | 5 |
| Mariano et al., 2019 | 0.5 | 0.5 | 1 | 1 | 1 | 4 |
| Ramanan et al., 2017 | 0.5 | 0.5 | 0 | 1 | 1 | 3 |
| Reyes et al., 2018 | 1 | 1 | 0.5 | 1 | 1 | 4.5 |
| Reyes et al., 2019 | 1 | 0.5 | 0.5 | 1 | 1 | 4 |
| Roca et al., 2013 | 0.5 | 1 | 0.5 | 1 | 1 | 4 |
| Russo et al., 2014 | 0.5 | 0.5 | 1 | 1 | 1 | 4 |
| Santamaria-García et al., 2016 | 0.5 | 1 | 1 | 1 | 1 | 4.5 |
| Santamaria-García et al., 2017 | 1 | 0.5 | 1 | 1 | 1 | 4.5 |
| Torralva et al., 2007 | 0.5 | 0.5 | 1 | 1 | 1 | 4 |
| Torralva et al., 2009 | 0.5 | 1 | 1 | 1 | 1 | 4.5 |
| Torralva et al., 2015 | 0.5 | 1 | 0.5 | 1 | 1 | 4 |
| Torralva et al., 2015 | 1 | 1 | 1 | 1 | 1 | 5 |
| Wajman et al., 2019 | 0.5 | 0 | 0 | 0.5 | 1 | 2 |
